# Novel Indicator to Ascertain the Status and Trend of COVID-19 Spread: Modeling Study

**DOI:** 10.2196/20144

**Published:** 2020-11-30

**Authors:** Takashi Nakano, Yoichi Ikeda

**Affiliations:** 1 Research Center for Nuclear Physics Osaka University Osaka Japan; 2 Department of Physics Faculty of Science Kyushu University Fukuoka Japan

**Keywords:** communicable diseases, COVID-19, SARS-CoV-2, model, modeling, virus, infectious disease, spread

## Abstract

**Background:**

In the fight against the pandemic of COVID-19, it is important to ascertain the status and trend of the infection spread quickly and accurately.

**Objective:**

The purpose of our study is to formulate a new and simple indicator that represents the COVID-19 spread rate by using publicly available data.

**Methods:**

The new indicator *K* is a backward difference approximation of the logarithmic derivative of the cumulative number of cases with a time interval of 7 days. It is calculated as a ratio of the number of newly confirmed cases in a week to the total number of cases.

**Results:**

The analysis of the current status of COVID-19 spreading over countries showed an approximate linear decrease in the time evolution of the *K* value. The slope of the linear decrease differed from country to country. In addition, it was steeper for East and Southeast Asian countries than for European countries. The regional difference in the slope seems to reflect both social and immunological circumstances for each country.

**Conclusions:**

The approximate linear decrease of the *K* value indicates that the COVID-19 spread does not grow exponentially but starts to attenuate from the early stage. The *K* trajectory in a wide range was successfully reproduced by a phenomenological model with the constant attenuation assumption, indicating that the total number of the infected people follows the Gompertz curve. Focusing on the change in the value of *K* will help to improve and refine epidemiological models of COVID-19.

## Introduction

The spread of COVID-19 has resulted in human and economic losses worldwide. To prevent the spread of the infection, it is sometimes necessary to restrict social activities by policies such as the blockade of cities and the prohibition of assembly. For the effective implementation of these policies, it is important to ascertain the status and trend of spread quickly and accurately. The purpose of our study is to formulate a simple indicator that represents the COVID-19 spread rate by using publicly available data [1-4] and to reveal the common features and regional differences of the spread. We also aim to check the validity of the widely accepted assumption that the number of daily confirmed cases can be approximated by the exponential function in the early stage of COVID-19 spread.

## Methods

To analyze the trend of COVID-19 spread, we introduced a new indicator *K* as a measure of the spread rate. It is defined by *K*(*t*) = 1 – *N*(*t* – 7)/*N*(*t*), where *t* is the number of days from a reference date and *N*(*t*) and *N*(*t* − 7) are the total number of cases on days *t* and (*t* − 7), respectively. Thus, the *K* value is a backward difference approximation of the logarithmic derivative of *N*(*t*) with a time interval of 7 days. If *N*(*t*) grows exponentially as obtained in the susceptible-infected-removed (SIR) model [5-10] during the early stage of the epidemic, *K* takes a constant value. For example, if *N*(*t*) keeps doubling in 7 days from the reference date, the corresponding *K* value is 0.5. Since *N*(*t*) is greater than *N*(*t* − 7) during the period from the initiation of spread to convergence, the range of the *K* value is between 0 and 1. We note that we can cancel the day of the week dependency seen in the number of daily confirmed cases by setting the interval to 7 days.

## Results

**Figure 1:**
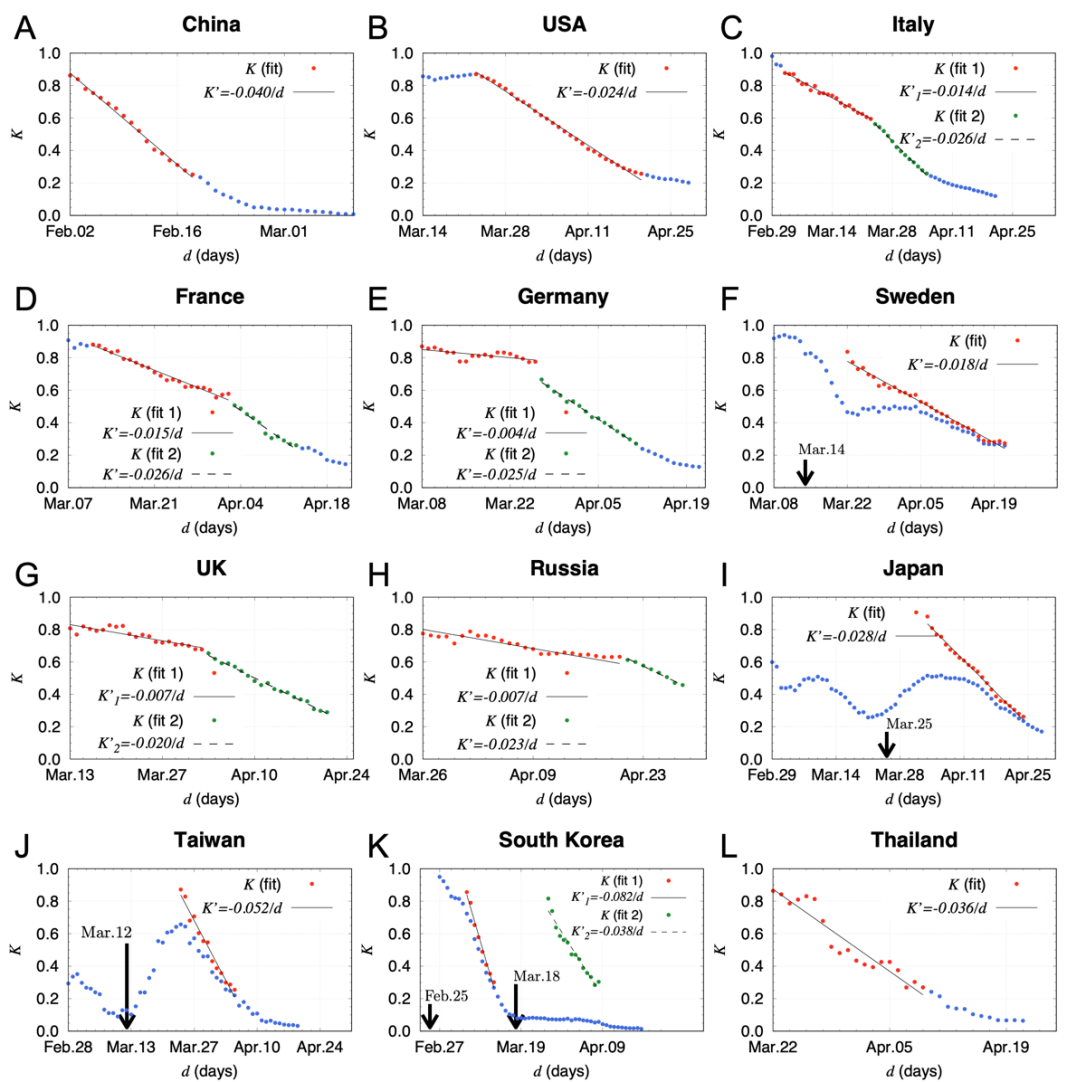
Transition of the *K* values from February to April 2020. (A) The *K* values of China obtained from the daily total number of infected people. The slope *K’* was obtained by a linear fit in the range of 0.25<*K*<0.9. The data points used for the fit are indicated by red points. The solid line is the fit result. (B) The *K* values of the United States. (C) The *K* values of Italy. The first and second *K’* values were obtained by linear fits using the red and green points, respectively. The solid line is the result of the first fit. The dashed line is the result of the second fit. (D) The *K* values of France. (E) The *K* values of Germany. (F) The *K* values of Sweden. The total number of infected people was counted from the reference date set for March 14. (G) The *K* values of the United Kingdom. (H) The *K* values of Russia. (I) The *K* values of Japan. The reference date was set for March 25. (J) The *K* values of Taiwan. The reference date was set for March 12. (K) The *K* values of South Korea. The reference dates were set for February 25 and March 18 for the first and second fits, respectively. (L) The *K* values of Thailand.

The *K* values for China were calculated by setting the reference date to January 26, 2020, where the data before February 12 were uniformly multiplied by 1.27 to correct a discontinuous increase in the number of infected people caused by the change of certification criteria for SARS-CoV-2 infections in Hubei Province on February 13. As shown in Figure 1, the trajectory of the *K* values is well approximated by a straight line with a slope (*K’*) of −0.0402 ± 0.0008/*d*. This linearly decreasing behavior evidently shows that the COVID-19 spread does not follow the exponential growth even in the early stage [8] but most likely follows a double exponential function known as the Gompertz curve (see the proceeding section, Interpretation of the *K* transition from a phenomenological model). The linearity of *K* is also prominent in the United States. After a high *K* level period indicating successive infectious explosions from mid- to late March, the *K* continued to decline with a uniform rate of *K’*=−0.0237 ± 0.0003/*d*.

Countries’ policies affected the transitions of the *K* values in Europe. In Italy, where COVID-19 started to spread first in Europe, *K’* was −0.0142 ± 0.0004/*d* from March 1 to March 23, 2020, indicating a slow pace of convergence, but the pace was improved after the implementation of a containment policy on March 24, resulting in *K’*=−0.0263 ± 0.0006/*d*. In France, where the spread started about 10 days after Italy, the slope *K’* was −0.0152 ± 0.0005/*d* for 3 weeks in the early stage, but it was improved to −0.0265 ± 0.0019/*d* after the lockdown. Germany began measures to prevent the spread of infection, such as closure of most retail stores on March 16, 2020, resulting in a steep slope (*K’*=−0.0252 ± 0.0005/*d*) afterward. Sweden, on the other hand, sought to acquire herd immunity and took relatively mild measures, resulting in a moderate slope (*K’*=−0.0179 ± 0.0004/*d*) lasting more than a month. The United Kingdom took similar measures in the early stage, resulting in infection explosion with *K’*=−0.0071 ± 0.0008/*d*. However, the slope became −0.0199 ± 0.0005/*d* after the introduction of stricter policies. In Russia, the *K* value stayed above 0.6 at *K’*=−0.0067 ± 0.0005/*d* until April 20 and then started to decrease at *K’*=−0.0234 ± 0.0010/*d*, indicating that a catastrophic situation was avoided.

In Asian countries, after the first wave originated from China, the subsequent spread worldwide caused the upward change in *K* trajectories. To measure the rate of the subsequent spread in terms of the *K* value accurately, the reference date had to be set at the rise of the spread. Thus, the *K* values for Japan were calculated by setting the reference date to March 25, 2020. The obtained slope (*K’*=−0.0283 ± 0.0006/*d*) was milder than those of Taiwan (*K’*=−0.0524 ± 0.0026/*d*) and South Korea (*K’*=−0.0820 ± 0.0042/*d* and *K’*=−0.0378 ± 0.0024/*d*), reflecting the difference in the strictness of countermeasures. However, it was steeper than those of European countries with more strict social restrictions than Japan. The relatively high absolute *K’* values even in the early stages were commonly observed in many Asian countries. For example, the *K’* value of Thailand is *K’*=−0.0361 ± 0.0028/*d*.

## Discussion

### Principal Findings and Interpretation of Results

As a new indicator of the COVID-19 spread rate, we have proposed the *K* value that takes a constant value if the number of infected people grows exponentially. The *K* values started to decrease even in the early stage of the COVID-19 spread, prior to the implementation of containment policies by the analyses of the real data for various countries. This suggests that the number of infected people does not follow the exponential growth. The analyses also revealed approximate linear decrease in the time evolution of the *K* value. The slope of the linear decrease differed from country to country. The characteristic steep slope in East and Southeast Asian countries in the early stage implies the existence of immunological factors [11,12] that suppressed the spread of COVID-19.

### Interpretation of the Linear *K* Transition From a Phenomenological Model

We have found that the *K* trajectory in the region of *K*<0.25 was reproduced by a phenomenological model with the constant attenuation assumption. If *N*(*t*) grows exponentially, the time evolution of *N*(*t*) is expressed as *N*(*t*) = exp(*at*)*N*(0), with a time-independent exponential constant *a*. However, the approximate linear decrease of the *K* value even in the early stage of the spread indicates that the constant *a* gradually decreased from the beginning. To introduce a small time dependence, we assumed that the constant *a* decreases exponentially, namely, *a*(*t*) = exp(–(1 – *k*)*t*) *a*(0), or equivalently, *a*(*t*) = exp(–(1 – *k*)) *a*(*t* – 1), with an attenuation factor *k* that is close to but less than 1. Under a condition of (1 – *k*)≪1, the time evolution of *a*(*t*) is approximated by *a*(*t*) = *k* *a*(*t* – 1) = *k^t^* *a*(0), which leads to *N*(*t* + 1) = exp(*k^t^* *a*(0))*N*(*t*). The model calculations under this constant attenuation assumption showed that the *K* trajectory can be approximated by a first order linear function of *t* in a wide range (0.25<*K*<0.9), with the slope *K’* being related to the attenuation factor *k* by the simple equation *k* = 1 + 2.88*K’* (see Multimedia Appendix 1). *N*(*t*) then follows the Gompertz curve [13,14], which is consistent with our constant attenuation assumption. It was also confirmed that the simple equation *k* = 1 + 2.88*K’* is derived analytically from the Taylor expansion at *K*=0.5 [13].

**Figure 2:**
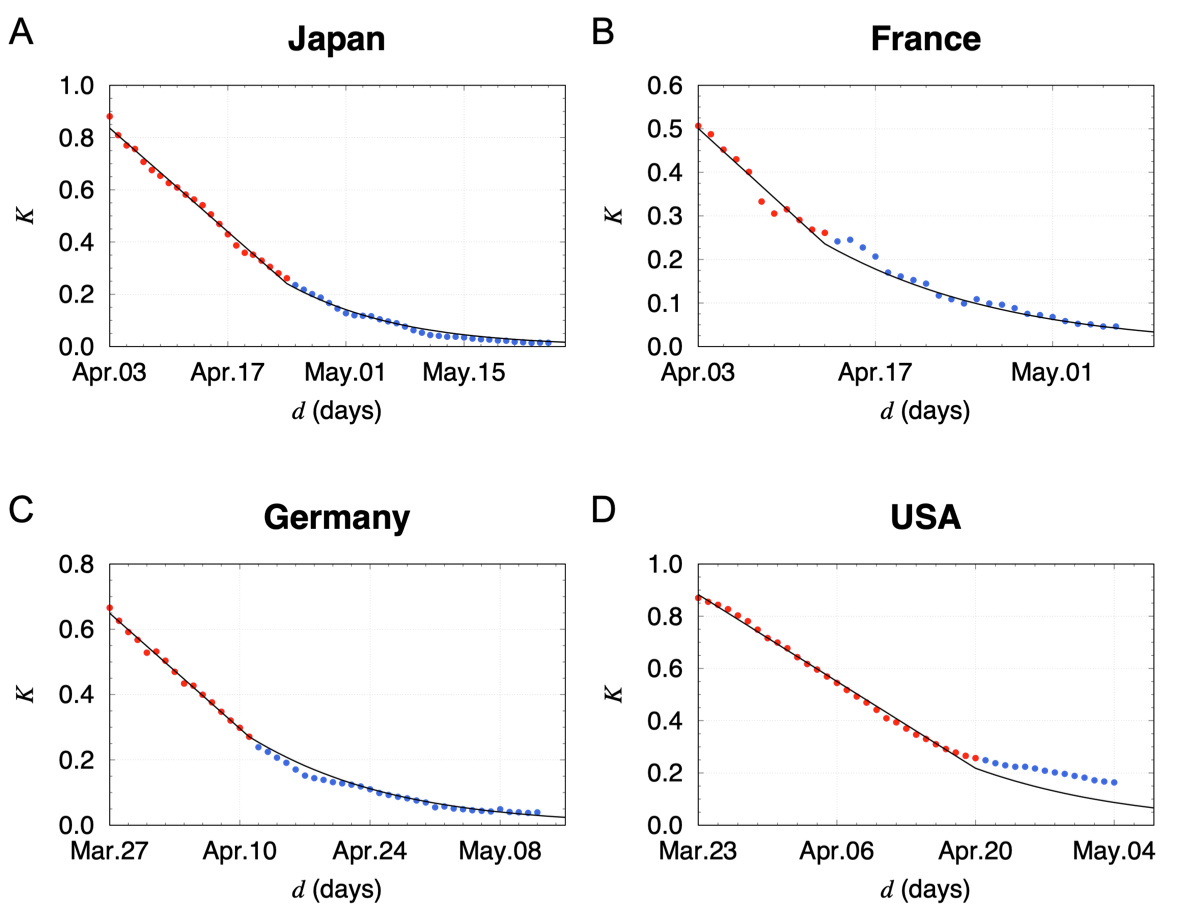
*K* values calculated by a phenomenological model with the constant attenuation assumption. (A) Model calculations and real data points for Japan. The slope *K’* was obtained by a linear fit in the range of 0.25<*K*<0.9 using red points. The solid curve in the range of *K*<0.25 is estimated with the constant attenuation factor *k* calculated from *K’*. The reference date was set for March 25, 2020. (B) Model calculations and real data points for France. (C) Model calculations and real data points for Germany. (D) Model calculations and real data points for the United States.

To test the long-term reliability and the validity of the assumption, we compared the model calculations with real data for Japan, France, Germany, and the United States. Using the *k* value calculated from *K’* obtained by a straight line fit in *K*>0.25, the *K* values below 0.25 were well reproduced by the model calculations for Japan, France, and Germany, as shown in Figure 2. This gives further indication that the total number of the infected people follows the Gompertz curve under fixed conditions for a long period. On the other hand, the data points started to deviate from the model calculation for the United States around April 18, 2020. The deviation was due to outbreaks in US states where COVID-19 spread was not serious in early April.

### Interpretation of the Linear *K* Transition From a Susceptible-Infected Epidemic Model

To understand the behavior of the *K* and the slope *K’*, we analyzed the public data of COVID-19 for the United States and Japan, employing the susceptible-infected (SI) model [10], which consists of the infected people (*I*) and susceptible people (*S* = *N* − *I*), with *N* being the final number of infected people. The SI model has the same time evolution of *I(t)* as the SIR model in the early stages of the epidemic. Both models predict the exponential growth of *I(t)*.

In the United States and Japan, several changes in the slope of the *K* were found; it is natural to extend the standard SI model to have several sources of COVID-19. The *K* is a monotonically decreasing function if a single source is taken into account like the standard SI model. The number of infected people with respect to each source *i* is described as:

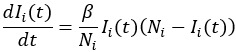



**Figure 3:**
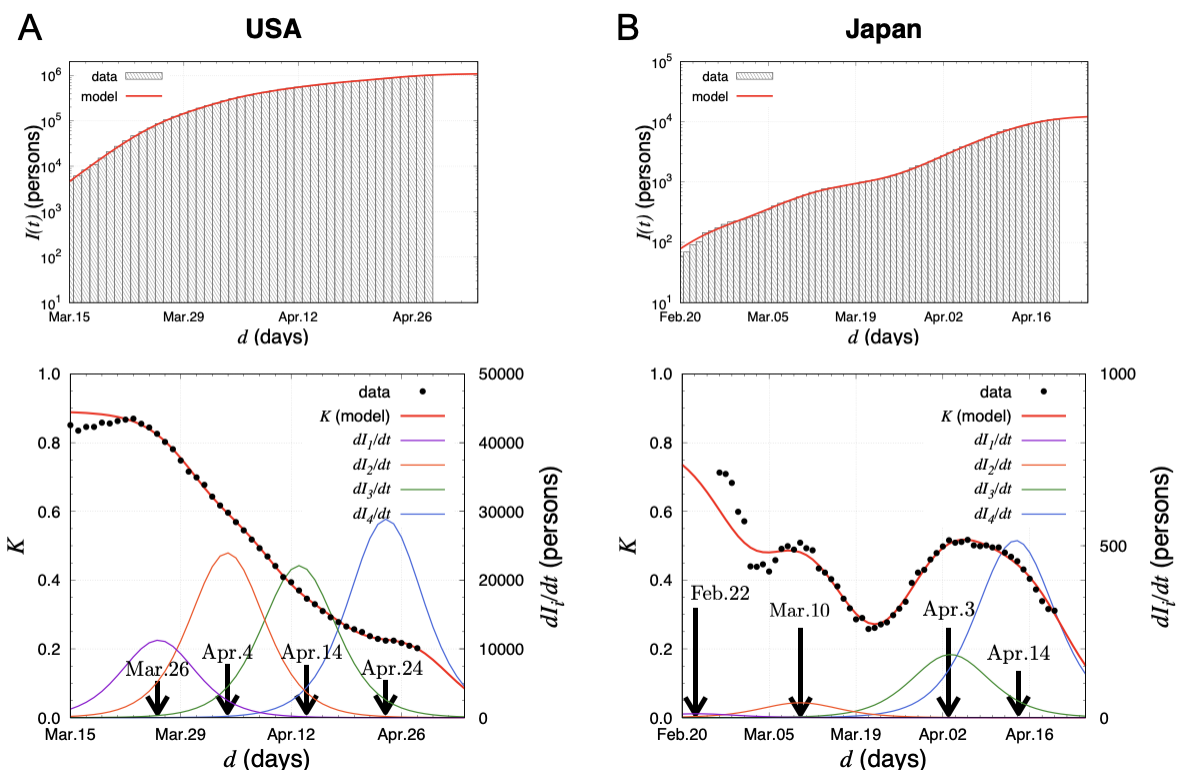
The model results of the total number of infected people and the *K* value in (A) the United States and (B) Japan. (A) The fit results are given in the top figure. The *K* value is represented together with the daily new cases *dI_i_/dt* calculated in the susceptible-infected model at the bottom. The peak positions tci caused by the source *i (i=1,2,3,4)* are shown by the vertical allows. (B) Same as (A) for the case of Japan. The reference date of February 19 was kept for the *K* values.

The model parameters *β* and *N_i_* control the spreading speed of COVID-19 and the final number of infected people caused by the source *i*, respectively. The analytic solution for *I_i_* is found as *I_i_*(*t*) = (*N_i_*/2) [1 + tanh(*β*/2(*t* − *t*^c^*_i_*))], with *t*^c^*_i_* denoting the peak time of the infection spreading. The total number of infected people in a country at time *t* are then obtained as 
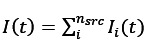
 and finally reach 
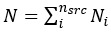
 As reported by Ranjan [15], each *N_i_* should be much smaller than the countries’ population sizes, so we chose *N_i_* as fit parameters. The optimal solution of the parameter set *P* = (*β,*{*N_i_*}*,*{*t*^c^*_i_*}) together with the number of sources *n*_src_ is found by minimizing the weighted mean squared deviation L(P) = ∑*_d_*_∈_*_D_*(*I*(*d*) – *N*(*d*))^2^/*N*(*d*) in the fit range *D*. Here, *N(d)* denotes the actual data of the total number of infected people on the day *d*. We chose *D* to be from February 23 to April 20, 2020 (58 days), for Japan and March 15 to April 28 (45 days) for the United States. We found the optimal number of sources for both countries as *n*_src_=4, and the resulting parameters are given by 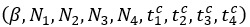 = (.32, 142k, 303k, 279k, 365k, 03/26, 04/04, 04/14, 04/24) for the United States and (.24, 0.2k, 0.7k, 3.1k, 8.7k, 02/22, 03/10, 04/03, 04/14) for Japan. Equipped with these parameters, the number of daily new cases *dI_i_/dt* and the *K* value are presented in Figure 3.

For the United States, the first peak position (March 26, 2020) clearly corresponded to the date of the change in the slope *K’*. This shows that the *K* starts to decrease when the first peak out takes place. The other sources contributed to keep the *K* linear as superposition. For Japan, there were two peaks in the actual data of the *K*, and they started to decrease on March 12 in the first peak and on April 11 in the second one. The peak positions in the model agree with these dates. Moreover, the third peak position in the model coincides with the date when the *K* saturates at 0.5. Comparing with the model analysis of the United States, it is worth mentioning that the parameter *β* was smaller in Japan than that in the United States. Nevertheless, the steep gradient of *K* was observed in Japan. This observation is understood by the deference of the number of infection sources contributing to the *K* in both countries. In Japan, the first two and last two sources are well separated in time so that only the last two affect the decrease of the *K* as superposition. However, in the United States, all four sources contributed to it. The results show that *K* plays a crucial role to understand how the infection spreads. The linearity of the *K* value is not trivial but is most likely to be caused by several consecutive infectious explosions.

### Conclusions

The analyses revealed that the time evolution of the *K* values in the region of 0.25<*K*<0.90 can be universally approximated by a straight line in many cases, indicating that the total number of infected people follows the Gompertz curve. This finding will be helpful to improve and refine epidemiological models of COVID-19. The slope *K’* of the straight line was different from country to country, and the regional difference in the slope seemed to reflect both social and immunological circumstances for each country. Focusing on the change in the value of *K* will help to improve and refine epidemiological models of COVID-19 and other infectious diseases with the same tendency as COVID-19.

## Appendix

Multimedia Appendix 1 Novel indicator of change in COVID-19 spread status.

## Abbreviations

SI: susceptible-infected
SIR: susceptible-infected-removed
