# Supplementary material for: Novel Indicator to Ascertain the Status and Trend of COVID-19 Spread: Modeling Study
Source: J Med Internet Res. 2020 Nov 30;22(11):e20144. doi: 10.2196/20144 (PMC7708296; doi:10.2196/20144)

**Novel indicator of change in COVID-19 spread status**

**Appendix**

**Phenomenological and susceptible-infected epidemic models**

The linear relation of *k* = 1 + 2*.*88*K’* was obtained from straight line fits of the *K* trajectories for the cases with *k* = 0*.*890, 0*.*895, 0*.*900, 0*.*905, 0*.*910, 0*.*915, 0*.*920, 0*.*925, 0*.*930, 0*.*935, 0*.*940 and 0*.*950 as shown in Figure 4. The maximum deviation of data points from the straight line is less than 11% of the data values in the fitting region of 0*.*25 *< K <* 0*.*9. We confirmed that the relation does not depend on the initial value *a*(0) as far as the first *K* value is larger than 0*.*9. Thus, we set *a*(0) to 0*.*5 for the all cases.

In epidemic models, the links of epidemic transmissions from infected persons to susceptible people grow exponentially with time. The constant attenuation assumption can be interpreted as follows: When the links of the transmissions are not partly connected to the susceptible people due to social and immunological circumstances, all the subsequent transmissions from unlinked susceptible people do not happen. Therefore, the number of possibly susceptible people decreases exponentially with time. In this interpretation, the constant attenuation factor *k* represents the rate of the partly connected links of COVID-19 transmissions.

**Figure 4.** Mock data of the *K* value and *K’* dependence of *k*.

(A) The attenuation factor *k* was assumed to be 0*.*95 (left), 0*.*920 (middle), and 0*.*890 (right). (B) *k* as a linear function of *K’* obtained by a fit with a constraint of *k* = 1 at *K’* = 0.


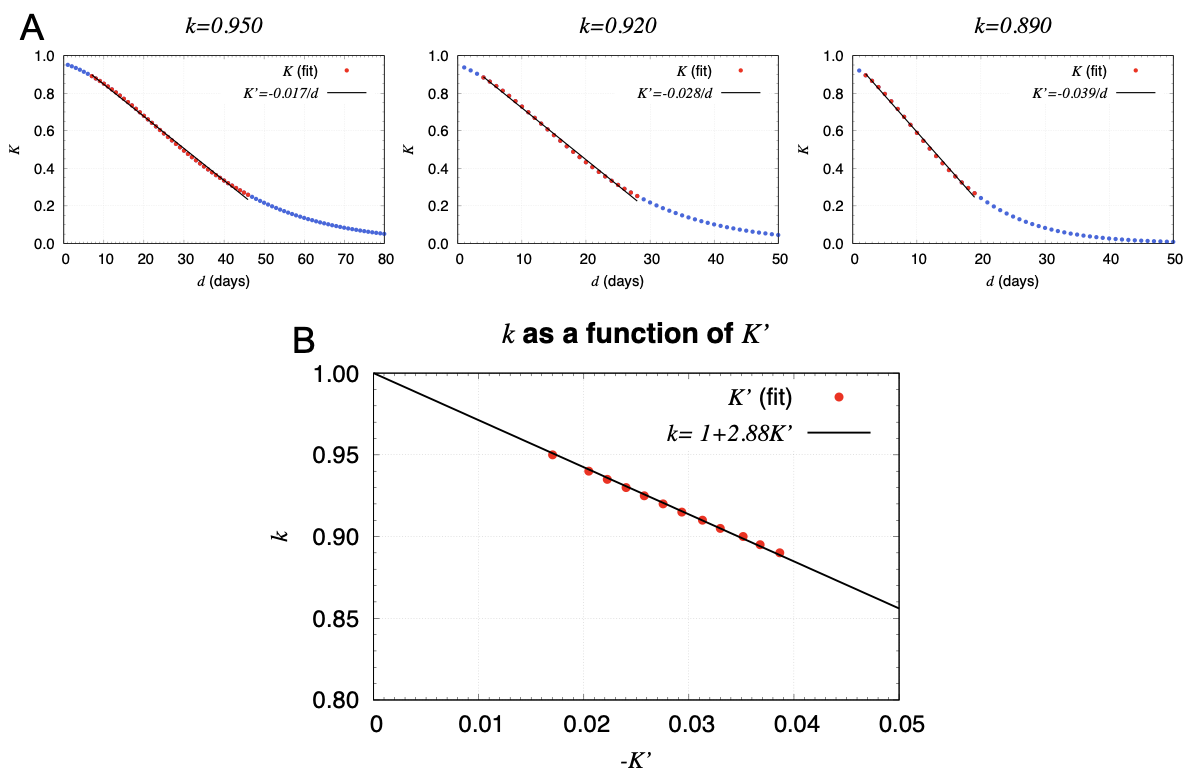

Supplement: Multimedia Appendix 1 [file jmir_v22i11e20144_app1.docx]
